# The Positive Role of Curcumin-Loaded Salmon Nanoliposomes on the Culture of Primary Cortical Neurons

**DOI:** 10.3390/md16070218

**Published:** 2018-06-25

**Authors:** Mahmoud Hasan, Shahrzad Latifi, Cyril J.F. Kahn, Ali Tamayol, Rouhollah Habibey, Elodie Passeri, Michel Linder, Elmira Arab-Tehrany

**Affiliations:** 1 Laboratoire d’Ingénierie des Biomolécules (LIBio), Université de Lorraine, 2 avenue de la Forêt de Haye—TSA 40602, 54518 Vandoeuvre CEDEX, France; mahmoud.hasan@univ-lorraine.fr (M.H.); cyril.kahn@univ-lorraine.fr (C.J.F.K.); elodie.passeri@univ-lorraine.fr (E.P.); michel.linder@univ-lorraine.fr (M.L.); 2 Department of Neurology, David Geffen School of Medicine, UCLA, Los Angeles, CA 90095, USA; SLatifi@mednet.ucla.edu; 3 Laboratory for Innovative Microtechnologies and Biomechanics, University of Nebraska-Lincoln, W332 NH, City Campus, Lincoln, NE 68588, USA; atamayol@unl.edu; 4 Department of Neuroscience and Brain Technologies, Istituto Italiano di Tecnologia, via Morego 30, 16163 Genova, Italy; Rouhollah.Habibey@iit.it

**Keywords:** nanoliposome, polar lipid, curcumin, transfer, primary cortical neurons

## Abstract

Curcumin (diferuloylmethane) is a natural bioactive compound with many health-promoting benefits. However, its poor water solubility and bioavailability has limited curcumin’s biomedical application. In the present study, we encapsulated curcumin into liposomes, formed from natural sources (salmon lecithin), and characterized its encapsulation efficiency and release profile. The proposed natural carriers increased the solubility and the bioavailability of curcumin. In addition, various physico-chemical properties of the developed soft nanocarriers with and without curcumin were studied. Nanoliposome-encapsulated curcumin increased the viability and network formation in the culture of primary cortical neurons and decreased the rate of apoptosis.

## 1. Introduction

**Figure 1:**
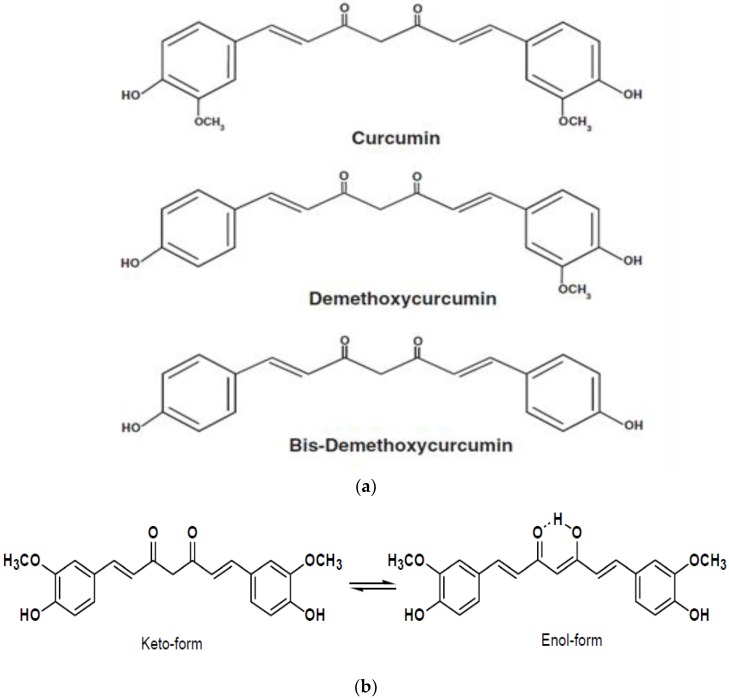
Chemical structure of curcuminoids (curcumin, demethoxycurcumin, bisdemethoxycurcumin) (**a**) and pH dependent keto- and enol- tautomeric form of curcumin (**b**).

Curcumin (CUR) is a hydrophobic polyphenolic compound derived from the rhizomes of Curcuma longa [1]. It is a derivative of turmeric, which is used as a natural spice in East Asian countries. Commercial curcumin consists of a mixture of three curcuminoids diferuloylmethane (~77%), demethoxycurcumin (~18%), and bisdemethoxycurcumin (~5%) [2] (Figure 1a). Curcumin is soluble in organic solvents, such as dimethylsulfoxide (DMSO), ethanol, or acetone, but its solubility in water is poor. In acidic and neutral conditions, the keto form predominates, and curcumin acts as a potent donor of H-atoms. But, under alkaline conditions (≥pH 8), the enolic form dominates, and the phenolic group of the molecule serves as an electron donor [3,4] (Figure 1b). Moreover, the data obtained by Barzegar (2012) suggest that in physiological condition, like inside the cells, where keto and enolic forms of curcumin are in the equilibrium, both of H-atom and electron transfer mechanisms are involved in decreasing free radicals [4].

Curcuminoids have become the focus of many research activities due to their numerous biological and pharmacological properties, such as antioxidant, anti-cancerous, anti-inflammatory, antimicrobial effects [5,6,7,8,9]. However, its poor water-solubility limits its uptake from the oral route as a food supplement [10]. Curcumin also has low bioavailability, which decreases its efficiency even if ingested [11]. Therefore, many attempts have been made to improve either curcumin’s water solubility or its bioavailability. These attempts involve the application of adjuvant-like piperine a known inhibitor of hepatic and intestinal glucuronidation [12], complexation with phospholipids and cyclodextrins, as well as the utilization of carriers in the form of liposomes, biodegradable microsphere, hydrogels, polymeric nanoparticles, and lipid based nanoparticles [10,11,13,14,15,16,17]. Achieving high encapsulation efficiency and gradual temporal release of curcumin are among the key challenges in exploitation of micro and nanocarriers [18,19]. Liposomes have been frequently employed in the literature as effective drug carriers. Liposomal carriers are formed from enclosed phospholipids membranes, which are amphiphilic molecules containing both water-soluble hydrophilic and lipid-soluble hydrophobic sections. Liposomes either encapsulate hydrophilic drugs within an aqueous core, or entrap lipophilic cargos within their lipid bilayer [20]. Liposomes can protect bioactive agents from digestion in the stomach and increase absorption rate in the gastrointestinal tract, leading to the enhancement of bioactivity and bioavailability [21]. Many liposomal formulations have been developed in recent years to improve the bioavailability of curcumin [22,23]. For example, the study of oral administration of liposome encapsulated curcumin in rats done by Takahashi and co-workers, showed high bioavailability and higher plasma concentration of curcumin [24].

The liposomal form of curcumin appears to be the best formulation to improve the bioavailability of curcumin in cells [25]. New forms of liposomes coated with propylene glycol, chitosan or protein have been developed, which reduce the release rate of curcumin and improve its bioaccessibility by promoting oral absorption [26,27,28].

Phospholipids, which are the main constituent of liposomes, are abundantly found in many tissues including nervous tissues and brain. This facilitates the delivery of liposomal drugs to the target tissues. Lecithin from salmon head (*Salmo salar*) contains a high percentage of polyunsaturated fatty acids (PUFA), such as eicosapentaenoic acid (EPA) and docosahexaenoic acid (DHA), which are important constituents of neuronal membranes [29,30]. The present study is primarily focused on the generation and physico-chemical characterizations of nanoliposomes from salmon lecithin as a carrier for hydrophobic drug, curcumin. Later, as a proof of principle, we studied the effect of the nanoliposome-encapsulated curcumin on neuronal network development using in vitro model of primary cortical neurons.

## 2. Results and Discussion

### 2.1. Fatty Acid Analyses

Salmon lecithin has higher variety of polyunsaturated fatty acids (PUFA) composition. Nine PUFAs of omega 3 and omega 6 were detected in salmon lecithin. The most significant proportions of fatty acids were C22:6 *n*-3 (28.15%) and C20:5 *n*-3 (8.83%). C18:1 *n*-9 and C16:0 were major monounsaturated and saturated fatty acids, respectively (19.11% and 19.33%). The ratio of *n*-3/*n*-6 was about 10.

### 2.2. Lipid Classes

The lipid classes of salmon lecithin showed that the phosphatidylcholine was a major class of phospholipids in salmon lecithin with 42%. Moreover, the percentage of triacylglycerols (TAG) in lecithin was 31.20 ± 0.4, and the percentage of polar fraction was 67.65 ± 0.9. Moreover, salmon lecithin contained a small amount of cholesterol, about 1.15 + 0.1%.

### 2.3. Liposome Size and Electrophoretic Mobility Measurements

**Figure 2:**
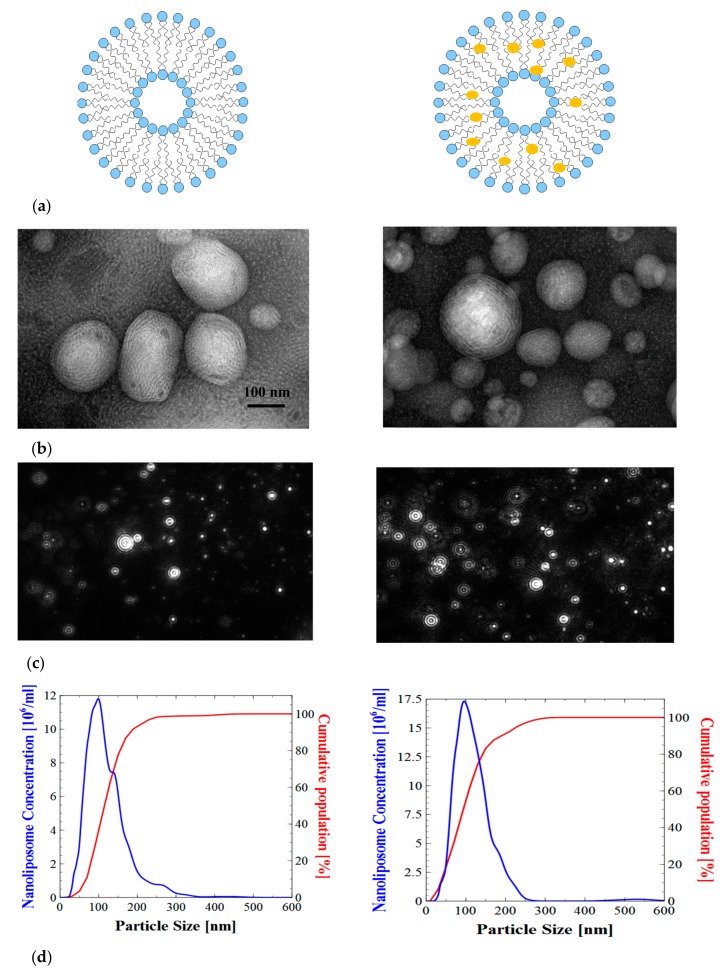
(**a**) Schematic of the study, demonstrating the nanoliposomes without (left) and with curcumin (right). (**b**) Transmission electron microscopic images of salmon nanoliposome before (left) and after curcumin encapsulation (right). (**c**) The size distribution of different nanoliposomes obtained from nanoparticle tracking analysis with the corresponding NTA video frame liposome (left), liposome loaded curcumin (right) and (**d**) size distribution from NTA liposome (left), liposome loaded curcumin (right).

The particle sizes of different nanoliposomes were measured immediately after sonication. The liposome size depends on the viscosity of the material and agitation parameters including sonication amplitude and time. The size of the nanoliposome also depends on fatty acid composition, lipid classes, and the surface-active properties of the lecithin [31,32]. The hydrodynamic diameter of nanoliposome for salmon lecithins was 119.97 ± 0.42 nm, and the polydispersity index was 0.31 ± 0.00. The size of the curcumin-loaded liposomes (114.37 ± 1.01 nm) was found to be slightly smaller than the unloaded curcumin liposomes. These, suggested a strong interaction between curcumin and lecithin that leads to the compaction of the core. Our results are in good agreement with the previously reported results [33]. Regarding polydispersity index, we observed no significant variation between curcumin loaded liposomes and unloaded liposomes (Figure 2). Electrophoretic mobility value was about −3.5 μm·cm/Vs with a relatively high stability, the polar fraction of lecithin (phosphate residues) had a negative charge. According to the Dynamic light Scatering (DLS) results, the electrophoretic mobility in salmon lecithins was −3.51 ± 0.11 μm·cm/Vs. Different types of phospholipids, like phosphatidylserine (PS), phosphatidic acid (PA), phosphatidylglycerol (PG), phosphatidylinositol (PI), phosphatidylethanolamine (PE), and phosphatidylcholine (PC) were present in salmon lecithin. Their charge was negative, except for PC and PE, which did not exhibit a net charge at physiological pH. Therefore, these anionic fractions were probably responsible for the negative electrophoretic mobility [34]. Nanoliposomes stability measured by particle size at 4 °C and 37 °C over one month, which showed no significant variation between day 0 and day 30.

The results obtained by Nanosight show clearly that the liposomes were not monodisperses, thus the spectra were multimodal explaining existence of many population of vesicle in the system, because the salmon lecithin contained various phospholipids with different types of fatty acids. Indeed, the mean diameter of particles obtained by NTA was highly similar to the Z-average values measured by DLS (Figure 2).

The small size of the nanoparticles could be exploited for increasing the absorption amount. Drugs delivered to mucosal surfaces are efficiently removed by mucus clearance mechanisms and systemic absorption, precluding prolong, and local drug presence. Encapsulation of drugs in polymeric particles offers the potential for localized and sustained delivery to mucosal tissues [35]. The mucus acts to entrap and remove pathogens and foreign particles, in order to protect the epithelial surface. Nanoparticles used in drug delivery are a good alternative to diffuse into the mucus layer and decrease elimination by clearance mechanism. In addition, the size of nanoparticles is an important parameter to cross the intestinal mucosal barrier, since the mesh-pore spacing of the mucus layer varies from 50–1800 nm [36]. Many studies have shown that nanoparticles with a size under 200 nm effectively diffuse through the mucus layer [37,38]. The liposomes smaller than 70 nm are removed from the systemic circulation by liver parenchymal cells, while those larger than 300 nm accumulate in the spleen. The highest blood concentration of liposomes is for the nanoparticles with size average of 70–200 nm [39,40]. 

The images obtained by Transmission Electron Microscopy (TEM) showed that nanoliposomes prepared by sonication method were in the form of multilamellar vesicles (MLV). The bilayer nature of the vesicles was clearly visible in these micrographs (Figure 2). We also observed some droplets in each formulation because of the presence of weak oil quantity (10%).

### 2.4. Entrapment Efficiency

High encapsulation efficiency is an important factor for the selection of a proper carrier and the encapsulation method. The entrapment efficiency of the curcumin in salmon liposome (2% *w*/*v*) was 56.2 ± 0.6%, which was low compared to the 67.3 ± 1.1%, observed in the 3% solution of salmon liposome [41]. This observation suggests that the entrapment efficiency was decreased as the ratio of curcumin to the lipid matrix increased, possibly due to the reduced available space for the drug to be entrapped.

**Figure 3:**
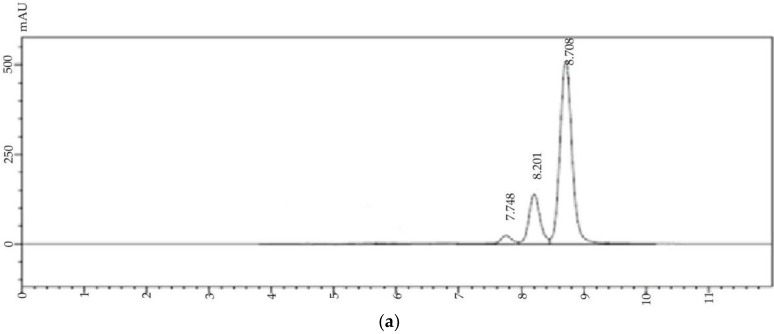

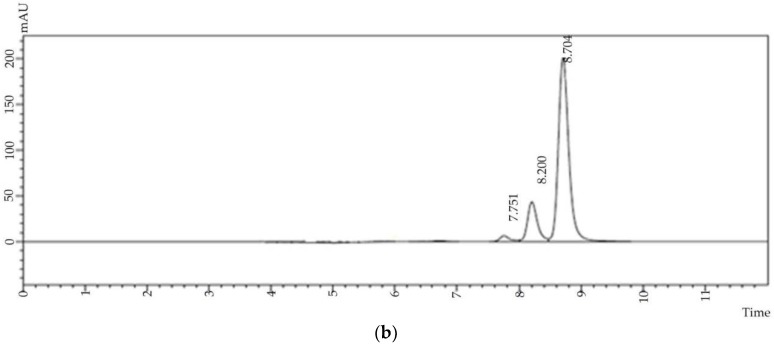
High-performance liquid chromatogram of (**a**) standard curcumin and (**b**) curcumin extracted from nanoliposomes.

Curcumin is a highly unstable molecule and its stability depends on various conditions such as light, pH, and temperature [42,43]. Curcumin retains its physiochemical properties and stability after the encapsulation in liposomes. The HPLC profile of curcumin (Figure 3a) showed the elution of this molecule at 8.7 min (78.2 ± 0.16%) along the demethoxycurcumin and bisdemethoxycurcumin at 8.02 min and 7.75 min (19.2 ± 0.1, and 2.6 ± 0.1, respectively). Extracted curcumin from nanoliposomes also showed a similar HPLC profile (Figure 3b).

### 2.5. Membrane Fluidity

Membrane fluidity, which reflects the order and dynamics of phospholipid alkyl chains, can affect the drug release profile from liposomes [29]. In fact, the drug leakage to the aqueous solution is enhanced by increasing the fluidity of the bilayer. Membrane fluidity depends on lipid composition of nanoliposomes. The presence of saturated FAs increases the packing between phospholipids, which expellees the water in the vicinity of the bilayer surface. On the other hand, unsaturated FAs help reducing the packing between phospholipids and preserve the level of hydration, thus maintaining membrane fluidity [44].

Nanoliposomes prepared from salmon lecithin had a membrane fluidity of 3.20 ± 0.03 when they contained higher proportion of polyunsaturated fatty acids. The lipid fluidity was expected to increase the permeant diffusion rate and partitioning tendency, which yielded a possible squaring effect on the enhancement factor.

In the presence of curcumin, membrane fluidity of liposome decreased (2.83 ± 0.1), which might be due to its molecular interaction with liposomes bilayer. Because, curcumin is a highly conjugated and rigid planar molecule, its presence can weaken hydrophobic interactions among acyl chains of phospholipids [45]. Curcumin incorporation perturbs the packing characteristics of the phospholipid bilayer and enhances the packing density of the hydrocarbon moiety in the lipid bilayer.

### 2.6. In Vitro Drug Release

**Figure 4:**
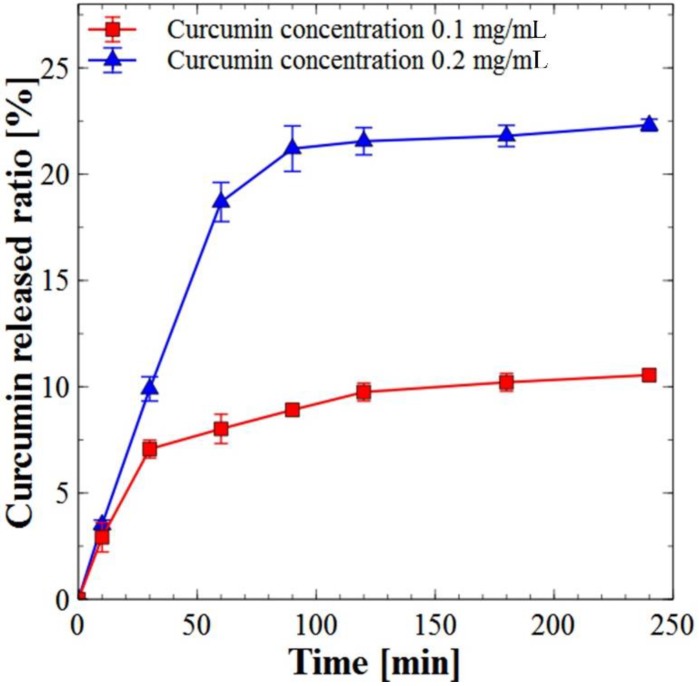
In vitro release of different concentrations of encapsulated curcumin from nanoliposome (values reported are mean ± SD; *n* = 3).

A biphasic release was observed for curcumin-loaded liposomes especially in the case of 0.2 mg/mL. The release profile showed a rapid release of about 18% within the first hour followed by sustained drug release of about 23% over 4 h. The drug entrapped within the bilayer of the liposome and high membrane fluidity might be the reason for the observed initial burst release, while the sustained release characteristics might be associated with the drug diffusion from the liposomes core (Figure 4).

The curcumin release from liposome towards the aqueous phase reached thermodynamic equilibrium after 2 h of incubation in the PBS solution, we continued up to 4 h to confirm that curcumin remained in liposome after 2 h. The kinetics of curcumin release could be explained by its higher affinity for lipid than aqueous phase [46]. Controlled curcumin release and delivery for longer duration can improve its bioavailability in an active and native form.

### 2.7. Neuronal Metabolic Capacity and Morphology

**Figure 5:**
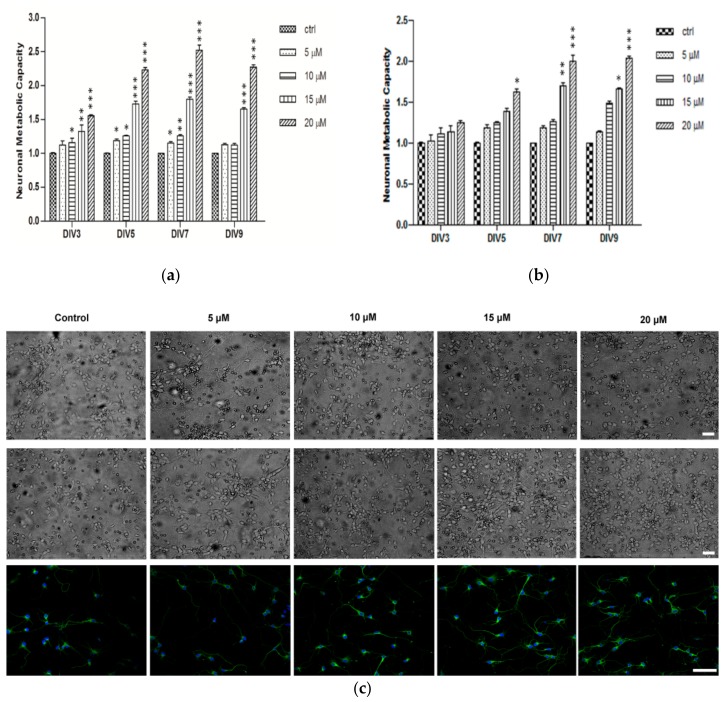
Curcumin increases the metabolic activity and the formation of neuronal network. Cortical neurons were incubated at different time-frame with the lecithin (**a**) or lecithin encapsulated curcumin (**b**) with indicated concentrations. Cell metabolic activity was checked by the XTT test. Results are expressed as normalized over control. (**c**) Phase contrast images of neuronal network cultures after treatment with lecithin or encapsulated curcumin at different concentration and immuno-fluorescent images of network formation from cortical neurons after treatment with curcumin. Data are mean ± standard error of three separate experiments from cells of different cultures. * *p* < 0.05, ** *p* < 0.01, *** *p* < 0.001.

Several studies demonstrated the importance of the nanoliposomes as natural carrier systems which are vastly applied as unique molecules in drug delivery strategies, owing to their unique characteristics [47,48]. In addition, multiple desirable characteristics of curcumin as a neuroprotective drug have been demonstrated, including anti-inflammatory, antioxidant, and anti-protein-aggregate activities [49,50,51]. Based on the originality of using natural source nanoliposomes for curcumin delivery, we treated primary cortical neurons with curcumin loaded salmon liposomes. To examine the effect of encapsulated curcumin on neuronal metabolic capacity, cortical neurons were assayed by XTT test at days 3, 5, 7, and 9 after treatment with 5, 10, 15 and 20 μM curcumin encapsulated nanoliposomes or nanoliposome alone. The doses were chosen based on our previous in vitro screening results [41]. All treatments were done 5 h after neuronal cell plating. Compared to the untreated control, the metabolic activity of treated neurons with higher concentrations (15 and 20 μM) was increased significantly from DIV3 to DIV9, and lower concentrations induced significant increase at DIV5 and DIV7 (Figure 5a). Treatment with nanoliposome alone did not show any statistically significant change at DIV3 and the maximum activity was only observed after DIV7 in highest concentration (Figure 5b). All these results indicated that curcumin-encapsulated nanoliposome induces higher metabolic activity of cortical neurons and has protective effect on their viability. In addition, qualitative evaluation of the bight-field and immunofluorescence microscopy images after 3DIV of treatment, confirmed increased neural network complexity in curcumin treated group. Morphological complexity of cortical network was achieved in higher encapsulated curcumin doses (15 and 20 μM, Figure 5c), whereas at lower concentration the network structure was more similar to the control cultures (Figure 5c). The congruent neuronal network activity increase (metabolic activity) and morphological complexity in high-dose curcumin treated cultures suggested that encapsulated curcumin improves in vitro network formation and development.

### 2.8. Encapsulated Curcumin Prevented Primary Cortical Neurons from Apoptosis

**Figure 6:**
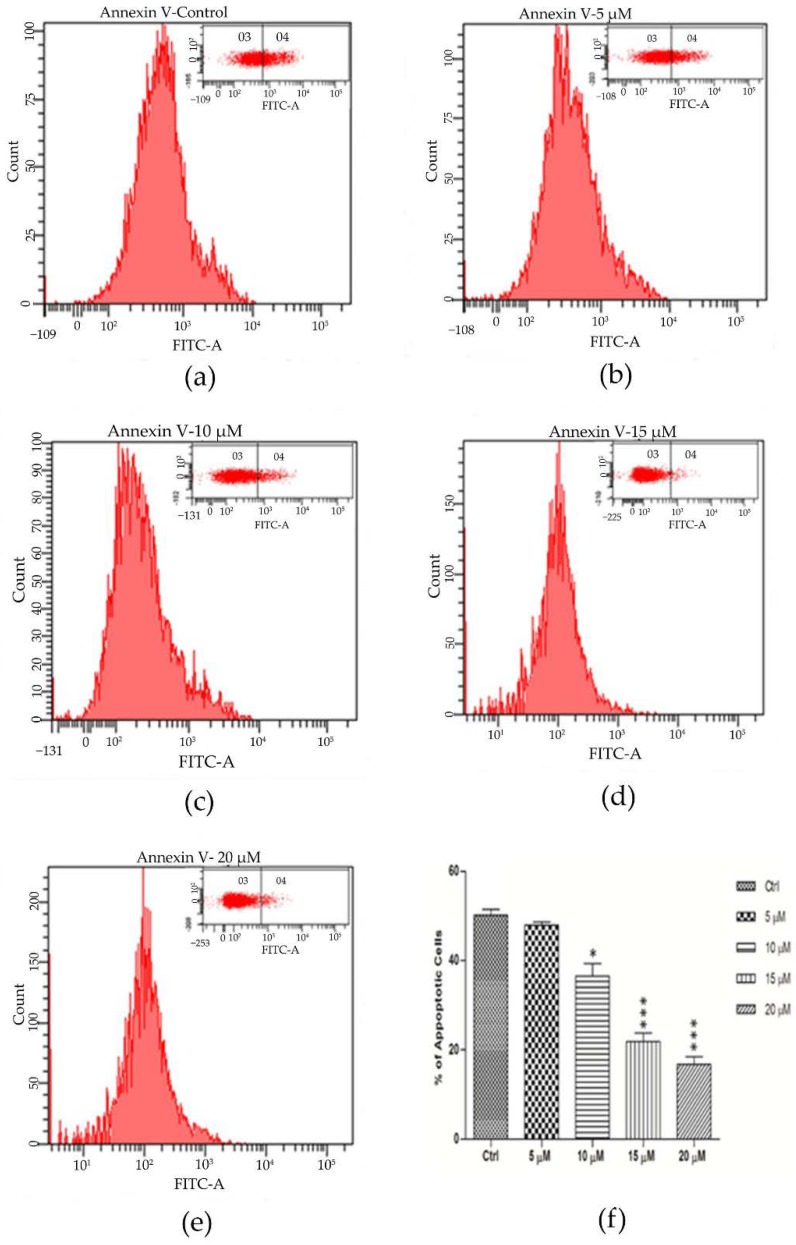
Curcumin encapsulated nanoliposom from natural lecithins decreases apoptosis in cortical neurons. (**a**–**e**) Primary cortical neurons were treated with 5, 10, 15, and 20 μM curcumin after 8 h for three days. Percentage of apoptotic cells was determined by annexin V-FITC fluorescence staining using flow cytometry analysis and fluorescence-activated cell sorting (FACS) analyzer. (**f**) Quantitation of these data represents the percentage of apoptotic cells in each condition. Data are expressed as mean ± Standard Error of the Mean (SEM) from three separate treatments.

Since curcumin promoted network elaboration and increased cellular metabolic activity, we studied the effect of encapsulated curcumin treatment on neuronal cell viability. Previous studies have shown the neuroprotective role of curcumin treatment [52,53]. We therefore undertook the current studies to determine whether curcumin encapsulated liposomes could protect neurons from apoptosis. To quantify the frequency of apoptotic cells, the cultures were incubated with FITC-labeled annexin V after treatment with different concentration of curcumin and were subsequently analyzed by flow cytometry. Figure 6a–e and in panel Q4 of the density plots (high level of annexin V-FITC) indicate a decrease in the percentage of apoptotic cells in the curcumin-treated cells compared with controls. In accordance with previous results, higher concentration of curcumin prevents neuronal apoptosis significantly compared to control (2.1 folds decrease for 15 μM and 2.7 folds for 20 μM), as was shown in Figure 6f. These data suggested that, curcumin promotes neuronal network metabolic activity and morphological complexity potentially via decreasing the neuronal cell apoptosis in the in vitro model.

## 3. Materials and Methods

Salmon lecithin was obtained by enzymatic hydrolysis in our laboratory as described before [54]. Lipidic fractions were extracted by a low temperature enzymatic process without utilization of solvents [54]. Following materials were used in the present study: Curcumin, acetonitrile and diethyl ether (Sigma-Aldrich, Saint-Quentin Fallavier, France), Boron trifluoride-methanol (BF_3_) (Bellefonte, PA, USA), Chloroform (VWR-Prolabo, Milan, Italy), Hexane, methanol and formic acid (Carlo-Erba, Val de Reuil, France), Ammoniac (Merck KGaA, Darmstadt, Germany). All the utilized organic solvents were analytical grade reagents.

### 3.1. Fatty Acids Composition

Fatty acid methyl esters (FAMEs) from salmon, soya and rapeseed lecithin were prepared as described by Ackman (1998) [55]. Then, FAMEs were analyzed using a Shimadzu 2010 gas chromatography (Shimadzu, Marne-la-Vallée, France) system equipped with a flame-ionization detector. Separation of FAME was accomplished on a fused silica capillary column (60 m, 0.25 mm i.d. × 0.20 µm film thicknesses, SPTM2380 Supelco, Bellefonte, PA, USA). Injector and detector temperatures were settled at 250 °C. The column temperature was fixed initially at 120 °C for 3 min, then raised to 180 °C at a rate of 2 °C min^−1^ and maintained at 220 °C for 25 min. Individual fatty acids were identified using Standard mixtures (PUFA1 from a marine source and PUFA2 from a vegetable source; Supelco, Sigma-Aldrich, Bellefonte, PA, USA). The results were shown as triplicate analyses.

### 3.2. Lipid Classes

The lipid classes of different fractions of lipid from salmon lecithin were determined by Iatroscan MK-5 TLC-FID (Iatron Laboratories Inc., Tokyo, Japan). The measurement was performed according to the protocol described in detail in our previous paper [41]. Two migrations were done to determine the proportion of neutral and polar lipid fractions. All standards were purchased from Sigma (Sigma-Aldrich Chemie GmbH, Taufkirchen, Germany). Area percentages of each pic were presented as the mean value of three repetitions.

### 3.3. Preparation of Nanoliposomes

One gram of lecithin and 10 mg curcumin were dissolved in ethanol, then a thin lipid film was formed on the wall of the flask by means of a Rotavapor in evaporating completely the organic solvent under vacuum, followed by hydration with 49 mL of distilled water and the suspension was agitated for 5 h under nitrogen. The samples were then sonicated at 40 KHz and 40% of full power for 360 s (1 s on, 1 s off) to obtain homogeneous solution. Liposome samples were stored in glass bottle in the dark at 37 °C.

### 3.4. Liposome Size and Electrophoretic Mobility Measurements

The mean size and electrophoretic mobility of liposomes were measured by dynamic light scattering (DLS) using a Malvern Zetasizer Nano ZS (Malvern Instruments Ltd., Malvern, UK). Prior to measuring size and electrophoretic mobility, the samples were diluted (1:200) with ultrapure distilled water. The size distribution of particle as well as the dispersed particles electrophoretic mobility was measured to evaluate the surface net charge around droplets. Measurements were made at 25 °C with a fixed scattering angle of 173°, the refractive index (RI) at 1.471 and absorbance at 0.01. The presented sizes were the z-average mean (dz) for the liposomal hydrodynamic diameter (nm). The measurements of electrophoretic mobility were performed in standard capillary electrophoresis cells equipped with gold electrodes at the same temperature. At least three independent measurements were performed for each condition.

### 3.5. Nanoparticle Tracking Analysis (NTA)

Vesicles were analyzed using a NanoSight LM10 instrument (NanoSight, Salisbury, UK). For this analysis, a monochromati laser beam at 405 nm was applied to the dilute suspension of vesicles. A video of 60 s duration was taken with a frame rate of 30 frames/s, and particle movement was analyzed using NTA software (version 2.1, NanoSight, Malvern Instruments Ltd., Malvern, UK). The NTA software is able to identify and track individual nanoparticles moving under Brownian motion and relates the velocity of particle movement to a particle size by applying the two dimensional Stokes-Einstein equation. The range of sizes that can be analyzed by NTA depends on the particle type, a high refractive index (e.g., colloidal gold) or a low refractive index (e.g., cell-derived vesicles). Exosomes and microvesicles have a low refractive index, and the smallest detectable size using the NTA system is approximately 50 nm. NTA post acquisition settings were optimized and kept constant between samples, and each video was then analyzed to give the mean, mode, and median vesicle size together with an estimate of the concentration. All experiments were carried out at 1:10,000 dilution and each measurement was performed in triplicate [56].

### 3.6. Stability of Nanoliposomes

The nanoliposomes containing curcumin and the control ones were stored in a drying-cupboard at 37 °C for 30 days. Mean particle size, electrophoretic mobility and polydispersity index of all formulations were analyzed every three days. The same protocol described previously was used for each analysis (Section 2.5 and Section 2.6).

### 3.7. Entrapment Efficiency of Curcumin

The percentage of drug incorporated was determined by centrifuging the drug-loaded nanoliposomes at 9000× *g* for 15 min to separate the unloaded curcumin crystals from the liposome. After centrifugation, an aliquot of liposomal dispersion was dissolved in 2 mL of methanol and the concentration of curcumin was assayed by HPLC at 425 nm after filtration through a membrane filter (0.2 µm) (Minisart^®^ RC15 Syringe Filters, Sartorius, Germany). The concentration of curcumin was determined by a reverse-phase HPLC system (Shimadzu, Kyoto, Japan) equipped with a quaternary pump (LC-20AD), an auto-injector (SIL-20AC HT), a UV-Vis photodiode array detector (UV-Vis PDA, SPD-M20A, Shimadzu, Marne-la-Vallée, France), a Zorbex SB-C18 column (5 µm, 4.6 mm × 250 mm) and Labsolution data software (Shimadzu, Marne-la-Vallée, France). Suspension was analyzed in isocratic mode using methanol (*v*/*v*, 5%), acetic acid 2% (*v*/*v*, 30%) and acetonitrile (*v*/*v*, 65%) at a flow rate of 0.5 mL·min^−1^. Aliquot (20 µL) was injected onto an Alltima^TM^ [HP C18, 5 µm (250 × 4.6 mm i.d.) column (GRACE, Deerfield, IL, USA)] at 25 °C. Detection of curcumin was performed at 425 nm after 8 min and 49 s. The experiments were performed in triplicate.

The encapsulation efficiency (EE) was calculated as:

(1) $$EE\left(\%\right)=\frac{Initial\;drug\;\left(g\right)-Free\;drug\left(g\right)}{Initial\;drug}\times 100.$$

### 3.8. Transmission Electron Microscopy (TEM)

Transmission electron microscopy was employed to observe the structure of nanliposomes and chitosan-coated liposome with a negative staining method according to the protocol of Colas et al. (2007) [57]. Briefly, the samples were diluted 25-folds with distilled water to reduce the concentration of the particles. Same volume of the diluted solution was mixed with an aqueous solution of ammonium molybdate (2%) as a negative staining agent. Staining was followed by a 3 min wait at room temperature, and 5 min on a copper mesh coated with carbon, then samples were examined using a Philips CM20 Transmission Electron Microscope associated with an Olympus TEM CCD camera.

### 3.9. Membrane Fluidity

Membrane fluidity of all samples was measured by fluorescence anisotropy measurements. TriMethylAmmonium DiPhenylHexatriene (TMA–DPH) was used as fluorescent probe which is a compound that contains a cationic TriMethylAmmonium (TMA) substitute that acts as a surface anchor to improve the localization of the fluorescent probe of membrane interiors, DiPhenylHexatriene (DPH). This measurement was carried out according to the method described by Maherani et al. [58]. Briefly, the solution of TMA–DPH (1 mM in ethanol) was added to the liposome suspension to reach finally a concentration of 4 µM and 0.2 mg/mL for the probe and the lipid, respectively. The mixture was lightly stirred for at least 1h at ambient conditions and protected from light. Then, 180 µL of the solution was distributed into each well of a 96-well black microplate. The fluorescent probe was vertically and horizontally oriented in the lipid bilayer. The fluorescent intensity of the samples was measured with Tescan INFINITE 200R PRO (Austria) equipped with fluorescent polarizers. Samples were excited at 360 nm and emission was recorded at 430 nm under constant stirring at 25 °C. The Magellan 7 software (Tecan group Ltd., Männedorf, Switzerland), was used for data analysis. The polarization value (*P*) of TMA–DPH was calculated using the following equation:

(2) $$P=\frac{I_{\mathrm{II}}-{\mathrm{GI}}_{┴}}{I_{\mathrm{II}}+2{\mathrm{GI}}_{┴}},$$

where I_II_ is the fluorescent intensity parallel to the excitation plane, I_⊥_ the fluorescent intensity perpendicular to the excitation plane, and G is the factor that accounts for transmission efficiency. Membrane fluidity is defined as 1/P. The results were presented as triplicate analyses.

### 3.10. In Vitro Drug Release

The percentage of curcumin released was determined as described earlier with suitable modification [59]. The 2% (*w*/*v*) solution of liposomes containing curcumin was dispersed in PBS (pH 7.4) at a ratio of 1:2. The dispersion was divided into 24 aliquots (1.8 mL each) in 2 mL Eppendorf tubes and they were kept at 37 °C. Due to low solubility of curcumin in aqueous solutions, released curcumin were forming crystals. At predetermined time intervals, the sample was centrifuged also at 9000× *g* for 10 min to separate the released curcumin crystals from the liposomal dispersion. After centrifugation, the concentration of curcumin was measured using a HPLC as described previously. The percentage of released curcumin was determined using the following equation.

(3) $$\mathrm{Drug}\;\mathrm{release}\left(\%\right)=\frac{\mathrm{Curcumin}\;\mathrm{released}\;\left(mg/mL\right)}{\mathrm{Curcumin}\;\mathrm{encapsulated}\;\left(mg/mL\right)}\times 100,$$

### 3.11. Quantification of Curcumin in Nanoliposomes

For in vitro quantification of curcumin, a standard solution of curcumin in methanol was prepared by adding 5 mg of curcumin into 1 mL of methanol solution. A serial dilution from 2 to 20 µg·mL^−1^ was done and examined at 425 nm using a UV spectrophotometer (Shimadzu UV-1605, Marne-la-Vallée, France) and an HPLC. The readings were drawn to generate a calibration curve to quantify the amount of drug in the nanoliposomes.

### 3.12. Embryonic Cortical Neurons Cell Culture

Primary cortical neurons were isolated from E18 Sprague Dawley rat embryos. All experiments were performed in accordance with the guideline established by European Community Council and were approved by the Italian Ministry of Health. The cortices were harvested in dissociation medium containing trypsin to digest tissues. Then, cortices were triturated. Neurons were seeded into six-well tissue culture plates, which were precoated with poly-d-lysine (Sigma, Saint-Quentin Fallavier, France). Neurons were maintained in Neurobasal Medium (Invitrogen, Carlsbad, CA, USA) supplemented with B27 (2 mL/100 mL, Invitrogen, Carlsbad, CA, USA), l-glutamine (1 mL/100 mL; Invitrogen, Carlsbad, CA, USA), and penicillin (50 U/mL)/streptomycin (50 μg/mL) in a 37 °C humidified incubator with a 5% CO_2_ in air. For biocompatibility test, neurons were directly plated on 96-well plates (15,000 cells per well) in neurobasal medium containing 2% B27 supplement, 2 mM glutamine and antibiotics.

### 3.13. XTT Test

The metabolic activity of the cortical neurons was assessed using the Cell Proliferation Kit II (XTT, 11 465 015 001, Roche, Mannheim, Germany) as per manufacturers protocol. Five hours after plating, neurons were treated with curcumin encapsulated nanoliposomes or nanoliposome alone at 5, 10, 15 and 20 μM concentrations or maintained in maintenance medium as non-treated control. Mitochondrial activity was measured at 3, 5, 7, and 9 days in vitro (DIV).

### 3.14. Immunofluorescence Assay

Cells were fixed with 4% PFA, 3% sucrose in PBS for 10 min at RT and permeabilized with 0.1%. Triton X-100 in PBS for 5 min at RT. Samples were blocked for 30 min in IF buffer (3% BSA, 2% goat serum in PBS). Cells were sequentially incubated with primary and secondary antibodies diluted in IF buffer (each for one hour at RT). Coverslips were mounted in Mowiol 4-88. Primary antibody was polyclonal anti-neuronal class III β-tubulin (#T2200, Sigma, Milan, Italy). Fluorescent-conjugated secondary antibody was from Molecular Probes (Invitrogen, Carlsbad, CA, USA). Images were acquired at an upright Leica TCS SP5 AOBS TANDEM confocal microscope equipped with a 60X/0.80 APO L W UVI objective (Leica Microsystems, Nanterre, France).

### 3.15. Flow Cytometry Analysis

Flow cytometry analyses were performed to measure cell apoptosis as previously described [60] using the annexin V-FITC apoptosis detection kit (#A9210, Sigma, Milan, Italy) following the manufacturer’s instructions. Primary cortical neurons were washed, briefly trypsinized, then washed again twice with cold 1× PBS. Harvested cells were then centrifuged and resuspended in 1× binding buffer. Cells were incubated with staining solution (annexin V-FITC) for 10 min in the dark at 4 °C. The cells were resuspended in 1× binding buffer before analyzed by flow cytometry. Samples were kept on ice during the entire procedure. Ten thousand cells from each sample were scanned and analyzed by FACS Calibur flow cytometry (Becton Dickinson, Franklin Lakes, NJ, USA) using the standard configuration and parameters. Finally, data acquisition and analysis was performed using Cell Quest software (Becton Dickinson, Franklin Lakes, NJ, USA).

### 3.16. Statistical Analysis

All data are presented as mean ± standard error. Analyses were carried out with ‘one-way ANOVA’ (for three or more groups) with Bonferroni corrections or student *t*-test (for two groups). Statistical significance was determined by Student’s *t*-tests with *p* values lower than 0.05.

## 4. Conclusions

The resemblance of liposomes to cellular membrane makes them a suitable carrier for the transportation of biomolecules through human tissues. The present study reported on the physico-chemical properties of nanoliposomes with and without curcumin encapsulation. The possibility of using liposomes as a suitable carrier system for different applications depends on their physico-chemical properties; fatty acid composition, and preparation methods strongly influence vesicle behavior in biological systems.

In general, particle size and z-potential are the most important properties that determine fate and stability of liposomes. Knowledge of the z-potential is also useful in controlling the aggregation, fusion and precipitation of liposomes, which are important factors affecting the stability of liposomal formulations. The lipid fluidity is expected to increase both the diffusivity and partitioning tendency of the permeant. Bilayer fluidity also reflects the order and dynamics of phospholipid alkyl chains in the bilayer and mainly depends on its composition, as liposomes composed of unsaturated lipids have more fluidity. The drug release characteristics exhibited controlled curcumins delivery for longer duration, which may improve bioavailability of the curcumins in its active, native form.

The results showed that salmon lecithin includes a wide range of fatty acids and specifically unsaturated ones EPA and DHA, which are known for their health benefits. From TEM imaging, we observed that lecithin solutions were largely made of nanoliposomes. Furthermore, the prepared salmon liposomes were stable for periods more than one month with polydisperse populations which were confirmed by Nanosight and zetasizer measurement.

Curcumin, a naturally occurring polyphenolic compound has been reported to exhibit several biological and pharmacological activities such as anti-tumor and anti-cancer properties [61,62,63]. Accumulating in vitro and in vivo data has shown that curcumin could act as strong candidate for prevention or treatment of neurodegenerative disorders like Parkinsons, Alzheimer’s, and stroke [64,65]. We previously demonstrated that nanoliposomes act as natural carrier systems have a positive role in neuronal metabolic activity and network formation. Application of nanoliposome-encapsulated curcumin in primary cortical neurons, demonstrated the effectiveness of the system in viability and formation of networks in comparison to untreated or only liposome treated cultures. The decrease in the apoptosis rate in treated cultures achieved in this study suggests the neuroprotective role of the engineered curcumin-loaded nanoliposomes. However, this hypothesis should be later tested in vivo and particularly in animal models of neurodegenerative disorders.
